# Bis[4′-(4-cyano­phen­yl)-2,2′:6′,2′′-terpyridine]cobalt(II) dichloride

**DOI:** 10.1107/S160053680904046X

**Published:** 2009-10-13

**Authors:** Kun Qian, Zhi-Hong Yan, Zhong-Wen Chen

**Affiliations:** a JiangXi University of Traditional Chinese Medicine, NanChang 330047, People’s Republic of China; b Key Laboratory of Modern Preparation of TCM, Ministry of Education of JiangXi, University of Traditional Chinese Medicine, NanChang 330047, People’s Republic of China

## Abstract

The title complex, [Co(C_22_H_14_N_4_)_2_]Cl_2_, has been synthesized by a solvothermal reaction of the 4′-(4-cyano­phen­yl)-2,2′:6′,2′′-terpyridine ligand with CoCl_2_·6H_2_O. The cobalt(II) ion is six-coordinated by two tridentate ligands in a distorted octa­hedral geometry. The benzene rings form dihedral angles of 30.02 (7) and 30.26 (7)° with the mean planes of the terpyridine ring systems. The chloride anions are statistically disordered over two positions with refined site occupancies of 0.601 (2) and 0.399 (2).

## Related literature

For the synthesis of functionalized terpyridines, see: Heller & Schubert (2003 ▶). For the structure of related cobalt complexes, see: Yu *et al.* (2008 ▶).
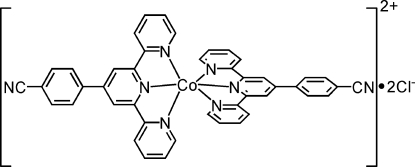


## Experimental

### 

#### Crystal data

[Co(C_22_H_14_N_4_)_2_]Cl_2_
*M* *_r_* = 798.57
Monoclinic, 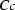

*a* = 13.258 (3) Å
*b* = 12.349 (3) Å
*c* = 25.394 (7) Å
β = 101.585 (13)°
*V* = 4072.9 (18) Å^3^
*Z* = 4
Mo *K*α radiation
μ = 0.59 mm^−1^
*T* = 291 K
0.30 × 0.26 × 0.24 mm

#### Data collection

Rigaku SCXmini diffractometer
Absorption correction: multi-scan (*CrystalClear*; Rigaku, 2005 ▶) *T*_min_ = 0.84, *T*_max_ = 0.87
18347 measured reflections
7941 independent reflections
7439 reflections with *I* > 2σ(*I*)
*R*_int_ = 0.030

#### Refinement

*R*[*F* ^2^ > 2σ(*F* ^2^)] = 0.041
*wR*(*F* ^2^) = 0.103
*S* = 1.04
7941 reflections
515 parameters
2 restraints
H-atom parameters constrained
Δρ_max_ = 0.19 e Å^−3^
Δρ_min_ = −0.32 e Å^−3^
Absolute structure: Flack (1983 ▶), 3940 Friedel pairs
Flack parameter: 0.079 (12)

### 

Data collection: *CrystalClear* (Rigaku, 2005 ▶); cell refinement: *CrystalClear*; data reduction: *CrystalClear*; program(s) used to solve structure: *SHELXS97* (Sheldrick, 2008 ▶); program(s) used to refine structure: *SHELXL97* (Sheldrick, 2008 ▶); molecular graphics: *SHELXTL* (Sheldrick, 2008 ▶); software used to prepare material for publication: *SHELXL97*.

## Supplementary Material

Crystal structure: contains datablocks I, global. DOI: 10.1107/S160053680904046X/rz2368sup1.cif

Structure factors: contains datablocks I. DOI: 10.1107/S160053680904046X/rz2368Isup2.hkl

Additional supplementary materials:  crystallographic information; 3D view; checkCIF report

## Figures and Tables

**Fig. 1.**
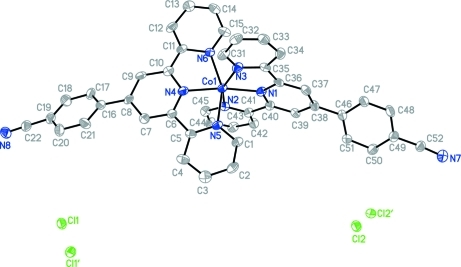
A view of the title compound with the atomic numbering scheme. Displacement ellipsoids are drawn at the 30% probability level. Hydrogen atoms are omitted for the clarity.

## supplementary crystallographic information

### Comment

Polypyridine ligands have played an important role in many areas. In particular, the chelating ligand terpyridine and its derivatives have been studied extensively as outstanding complexing agents for a wide range of metal ions (Heller & Schubert, 2003). In this paper, we report the crystal structure of the title compound obtained by a solvothermal reaction of CoCl_2_.6H_2_O and the tridentate 4'-(4-cyanophenyl)-2,2': 6',2''-terpyridine ligand.

An *ORTEP* drawing of the title compound is shown in Fig. 1. Both Cl^-^ anions are disordered over two positions with refined site occupancies of 0.601 (2) and 0.399 (2). The cobalt(II) ion is six-coordinated by two *mer*-arranged tridentate N_3_-terpyridine ligands in a distorted octahedral geometry. As observed in the related complexes bis[4'-(4-cyanophenyl)-2,2':6',2''-terpyridine]cobalt(II) bis(tetrafluoridoborate) nitromethane solvate and bis[4'-(4-cyanophenyl)-2,2':6',2''-terpyridine]cobalt(III) tris(tetrafluoridoborate) nitromethane solvate (Yu *et al.*, 2008), the Co–N1 and Co–N4 bond distances of the central pyridine rings [2.000 (3) and 1.980 (3) Å, respectively] are significantly shorter than those involving the side pyridine rings [Co–N2 = 2.105 (3) Å; Co–N3 = 2.089 (3) Å; Co–N5 = 2.119 (3) Å; Co–N6 = 2.093 (3) Å], resulting in a pronounced distortion of the octahedral coordination geometry at the metal centre. The terpyridine ring systems are only approximately planar (maximum deviation of 0.170 (4) Å for atom C34) and form dihedral angles of 30.02 (7) and 30.26 (7)° with the attached benzene rings.

### Experimental

A mixture of 4'-(4-cyanophenyl)-2,2':6',2''-terpyridine (33.4 mg, 0.1 mmol), CoCl_2_.6H_2_O (12 mg, 0.05 mmol) in a 10% water-ethanol solution (2 ml) were sealed in a glass tube was kept at 125 °C. Red crystals suitable for X-ray analysis were obtained after 5 days.

### Refinement

All H atoms were positioned geometrically and treated as riding, with C—H = 0.93 Å and *U*_iso_(H) = 1.2 *U*_eq_(C).

### Crystal data

**Table:**

[Co(C_22_H_14_N_4_)_2_]Cl_2_	*F*(000) = 1636
*M**_r_* = 798.57	*D*_x_ = 1.302 Mg m^−^^3^
Monoclinic, *C**c*	Mo *K*α radiation, λ = 0.71073 Å
Hall symbol: C -2yc	Cell parameters from 2346 reflections
*a* = 13.258 (3) Å	θ = 2.1–27.4°
*b* = 12.349 (3) Å	µ = 0.59 mm^−^^1^
*c* = 25.394 (7) Å	*T* = 291 K
β = 101.585 (13)°	Block, red
*V* = 4072.9 (18) Å^3^	0.30 × 0.26 × 0.24 mm
*Z* = 4	

### Data collection

**Table:**

Rigaku SCXmini diffractometer	7941 independent reflections
Radiation source: sealed tube	7439 reflections with *I* > 2σ(*I*)
graphite	*R*_int_ = 0.030
Detector resolution: 13.6612 pixels mm^-1^	θ_max_ = 26.0°, θ_min_ = 3.0°
ω scans	*h* = −16→16
Absorption correction: multi-scan (*CrystalClear*; Rigaku, 2005)	*k* = −15→15
*T*_min_ = 0.84, *T*_max_ = 0.87	*l* = −31→31
18347 measured reflections	

### Refinement

**Table:**

Refinement on *F*^2^	Secondary atom site location: difference Fourier map
Least-squares matrix: full	Hydrogen site location: inferred from neighbouring sites
*R*[*F*^2^ > 2σ(*F*^2^)] = 0.041	H-atom parameters constrained
*wR*(*F*^2^) = 0.103	*w* = 1/[σ^2^(*F*_o_^2^) + (0.06*P*)^2^ + 1.99*P*] where *P* = (*F*_o_^2^ + 2*F*_c_^2^)/3
*S* = 1.04	(Δ/σ)_max_ < 0.001
7941 reflections	Δρ_max_ = 0.19 e Å^−^^3^
515 parameters	Δρ_min_ = −0.32 e Å^−^^3^
2 restraints	Absolute structure: Flack (1983), 3940 Friedel pairs
Primary atom site location: structure-invariant direct methods	Flack parameter: 0.079 (12)

### Special details

**Table:**

Geometry. All e.s.d.'s (except the e.s.d. in the dihedral angle between two l.s. planes) are estimated using the full covariance matrix. The cell e.s.d.'s are taken into account individually in the estimation of e.s.d.'s in distances, angles and torsion angles; correlations between e.s.d.'s in cell parameters are only used when they are defined by crystal symmetry. An approximate (isotropic) treatment of cell e.s.d.'s is used for estimating e.s.d.'s involving l.s. planes.
Refinement. Refinement of *F*^2^ against ALL reflections. The weighted *R*-factor *wR* and goodness of fit *S* are based on *F*^2^, conventional *R*-factors *R* are based on *F*, with *F* set to zero for negative *F*^2^. The threshold expression of *F*^2^ > σ(*F*^2^) is used only for calculating *R*-factors(gt) *etc*. and is not relevant to the choice of reflections for refinement. *R*-factors based on *F*^2^ are statistically about twice as large as those based on *F*, and *R*- factors based on ALL data will be even larger.

### Fractional atomic coordinates and isotropic or equivalent isotropic displacement parameters (Å^2^)

**Table:**

	*x*	*y*	*z*	*U*_iso_*/*U*_eq_	Occ. (<1)
C1	0.4556 (2)	0.7500 (3)	0.66226 (14)	0.0433 (7)	
H1A	0.4430	0.7713	0.6955	0.052*	
C2	0.4258 (3)	0.6489 (3)	0.64410 (15)	0.0504 (8)	
H2A	0.3967	0.6013	0.6653	0.060*	
C3	0.4396 (3)	0.6178 (3)	0.59307 (15)	0.0533 (9)	
H3A	0.4179	0.5498	0.5797	0.064*	
C4	0.4871 (3)	0.6904 (3)	0.56123 (15)	0.0525 (8)	
H4A	0.4986	0.6711	0.5275	0.063*	
C5	0.5151 (2)	0.7915 (2)	0.58384 (13)	0.0390 (6)	
C6	0.5656 (2)	0.8774 (2)	0.55783 (12)	0.0394 (6)	
C7	0.5987 (3)	0.8635 (3)	0.50908 (15)	0.0517 (8)	
H7A	0.5854	0.7988	0.4902	0.062*	
C8	0.6519 (2)	0.9472 (3)	0.48857 (12)	0.0400 (7)	
C9	0.6644 (3)	1.0457 (2)	0.51690 (14)	0.0439 (7)	
H9A	0.6953	1.1039	0.5030	0.053*	
C10	0.6306 (2)	1.0582 (3)	0.56667 (13)	0.0404 (7)	
C11	0.6467 (2)	1.1512 (2)	0.59883 (13)	0.0391 (6)	
C12	0.6815 (3)	1.2479 (3)	0.58696 (15)	0.0555 (9)	
H12A	0.7020	1.2584	0.5544	0.067*	
C13	0.6872 (3)	1.3370 (3)	0.62514 (15)	0.0522 (8)	
H13A	0.7102	1.4049	0.6169	0.063*	
C14	0.6589 (3)	1.3198 (3)	0.67199 (15)	0.0543 (9)	
H14A	0.6594	1.3764	0.6962	0.065*	
C15	0.6290 (3)	1.2182 (3)	0.68424 (16)	0.0514 (8)	
H15A	0.6148	1.2075	0.7183	0.062*	
C16	0.6931 (3)	0.9314 (3)	0.43904 (13)	0.0423 (7)	
C17	0.7813 (3)	0.9872 (3)	0.43298 (14)	0.0543 (9)	
H17A	0.8097	1.0391	0.4581	0.065*	
C18	0.8274 (3)	0.9655 (3)	0.38933 (17)	0.0597 (9)	
H18A	0.8870	1.0020	0.3857	0.072*	
C19	0.7838 (3)	0.8889 (3)	0.35105 (14)	0.0554 (9)	
C20	0.6954 (3)	0.8334 (4)	0.35633 (15)	0.0593 (10)	
H20A	0.6667	0.7824	0.3307	0.071*	
C21	0.6496 (3)	0.8545 (3)	0.40035 (16)	0.0553 (9)	
H21A	0.5902	0.8176	0.4040	0.066*	
C22	0.8327 (3)	0.8633 (3)	0.30799 (15)	0.0565 (9)	
C31	0.3510 (2)	1.0851 (3)	0.62015 (14)	0.0471 (8)	
H31A	0.3687	1.0861	0.5865	0.057*	
C32	0.2585 (2)	1.1288 (3)	0.62426 (14)	0.0478 (8)	
H32A	0.2171	1.1625	0.5949	0.057*	
C33	0.2299 (3)	1.1225 (3)	0.66953 (14)	0.0488 (8)	
H33A	0.1655	1.1492	0.6722	0.059*	
C34	0.2927 (2)	1.0764 (3)	0.71530 (14)	0.0450 (7)	
H34A	0.2732	1.0734	0.7484	0.054*	
C35	0.3859 (3)	1.0358 (2)	0.70731 (14)	0.0418 (7)	
C36	0.4629 (2)	0.9833 (2)	0.75215 (13)	0.0388 (7)	
C37	0.4442 (2)	0.9479 (3)	0.80124 (13)	0.0422 (7)	
H37A	0.3811	0.9625	0.8104	0.051*	
C38	0.5186 (2)	0.8908 (3)	0.83676 (13)	0.0423 (7)	
C39	0.6133 (2)	0.8692 (3)	0.82105 (11)	0.0370 (6)	
H39A	0.6655	0.8318	0.8438	0.044*	
C40	0.6266 (2)	0.9048 (3)	0.77123 (12)	0.0383 (6)	
C41	0.7216 (2)	0.8845 (3)	0.74866 (12)	0.0393 (6)	
C42	0.8132 (2)	0.8443 (3)	0.77910 (14)	0.0468 (7)	
H42A	0.8195	0.8252	0.8150	0.056*	
C43	0.8944 (2)	0.8347 (3)	0.75235 (13)	0.0455 (7)	
H43A	0.9577	0.8109	0.7716	0.055*	
C44	0.8859 (3)	0.8579 (3)	0.70063 (14)	0.0457 (7)	
H44A	0.9408	0.8466	0.6835	0.055*	
C45	0.7900 (2)	0.9006 (3)	0.67171 (14)	0.0458 (7)	
H45A	0.7841	0.9200	0.6358	0.055*	
C46	0.4954 (3)	0.8463 (3)	0.88779 (13)	0.0443 (7)	
C47	0.4241 (3)	0.8945 (3)	0.91366 (13)	0.0470 (7)	
H47A	0.3922	0.9584	0.8998	0.056*	
C48	0.3989 (3)	0.8503 (3)	0.95938 (14)	0.0566 (10)	
H48A	0.3524	0.8845	0.9768	0.068*	
C49	0.4469 (3)	0.7497 (3)	0.97918 (14)	0.0494 (8)	
C50	0.5180 (3)	0.7026 (3)	0.95501 (16)	0.0543 (8)	
H50A	0.5498	0.6386	0.9687	0.065*	
C51	0.5438 (3)	0.7506 (3)	0.90896 (14)	0.0451 (7)	
H51A	0.5931	0.7185	0.8927	0.054*	
C52	0.4167 (3)	0.7051 (3)	1.02482 (13)	0.0497 (8)	
Cl1	0.72405 (11)	0.45349 (12)	0.35812 (5)	0.0485 (4)	0.601 (2)
Cl2	0.56620 (11)	0.38709 (12)	0.92594 (5)	0.0514 (4)	0.601 (2)
Cl1'	0.81405 (18)	0.33040 (19)	0.38569 (9)	0.0536 (6)	0.399 (2)
Cl2'	0.64297 (16)	0.43338 (17)	0.98097 (9)	0.0480 (5)	0.399 (2)
Co1	0.56329 (2)	0.97563 (3)	0.660571 (17)	0.03465 (10)	
N1	0.5537 (2)	0.9571 (2)	0.73767 (12)	0.0383 (6)	
N2	0.70937 (18)	0.9132 (2)	0.69502 (10)	0.0395 (6)	
N3	0.4179 (2)	1.0409 (2)	0.66062 (11)	0.0436 (6)	
N4	0.5838 (3)	0.9701 (2)	0.58551 (13)	0.0459 (7)	
N5	0.50297 (19)	0.8216 (2)	0.63437 (11)	0.0374 (5)	
N6	0.6186 (2)	1.1312 (2)	0.65007 (11)	0.0432 (6)	
N7	0.3920 (2)	0.6612 (3)	1.06197 (12)	0.0552 (7)	
N8	0.8747 (3)	0.8401 (3)	0.27126 (13)	0.0613 (8)	

### Atomic displacement parameters (Å^2^)

**Table:**

	*U*^11^	*U*^22^	*U*^33^	*U*^12^	*U*^13^	*U*^23^
C1	0.0369 (15)	0.0384 (16)	0.0550 (18)	−0.0097 (12)	0.0103 (13)	0.0049 (14)
C2	0.0429 (18)	0.0469 (19)	0.064 (2)	−0.0191 (15)	0.0168 (15)	0.0096 (16)
C3	0.0385 (17)	0.059 (2)	0.060 (2)	−0.0194 (15)	0.0027 (15)	−0.0081 (17)
C4	0.052 (2)	0.0401 (18)	0.061 (2)	−0.0016 (14)	0.0011 (16)	−0.0100 (15)
C5	0.0376 (15)	0.0299 (14)	0.0463 (16)	0.0047 (12)	0.0003 (12)	−0.0015 (12)
C6	0.0455 (17)	0.0322 (15)	0.0377 (15)	−0.0036 (12)	0.0013 (12)	0.0028 (12)
C7	0.063 (2)	0.0440 (18)	0.0527 (19)	−0.0052 (16)	0.0228 (17)	−0.0014 (15)
C8	0.0460 (17)	0.0369 (15)	0.0314 (14)	0.0031 (13)	−0.0054 (12)	0.0138 (12)
C9	0.0508 (18)	0.0262 (14)	0.0557 (19)	0.0024 (12)	0.0129 (15)	0.0108 (13)
C10	0.0301 (14)	0.0406 (16)	0.0472 (16)	−0.0022 (12)	0.0000 (12)	0.0068 (13)
C11	0.0328 (14)	0.0344 (15)	0.0482 (16)	0.0037 (12)	0.0035 (12)	0.0125 (13)
C12	0.067 (2)	0.0419 (19)	0.055 (2)	−0.0191 (17)	0.0046 (17)	0.0135 (15)
C13	0.0383 (17)	0.0499 (19)	0.067 (2)	−0.0189 (15)	0.0076 (15)	−0.0020 (17)
C14	0.056 (2)	0.0464 (19)	0.060 (2)	−0.0027 (16)	0.0096 (17)	−0.0196 (16)
C15	0.0401 (17)	0.0428 (19)	0.071 (2)	0.0005 (14)	0.0094 (15)	−0.0177 (16)
C16	0.0464 (16)	0.0396 (16)	0.0386 (15)	0.0070 (13)	0.0030 (13)	0.0065 (13)
C17	0.069 (2)	0.056 (2)	0.0392 (16)	−0.0119 (17)	0.0138 (16)	0.0093 (15)
C18	0.057 (2)	0.063 (2)	0.064 (2)	0.0085 (17)	0.0223 (18)	−0.0002 (18)
C19	0.067 (2)	0.058 (2)	0.0415 (17)	0.0193 (18)	0.0111 (16)	0.0131 (16)
C20	0.071 (3)	0.067 (2)	0.0409 (18)	0.013 (2)	0.0142 (17)	0.0030 (17)
C21	0.061 (2)	0.048 (2)	0.059 (2)	−0.0084 (17)	0.0161 (17)	−0.0154 (17)
C22	0.065 (2)	0.056 (2)	0.051 (2)	0.0216 (18)	0.0165 (16)	0.0117 (16)
C31	0.0339 (15)	0.0512 (19)	0.0505 (18)	0.0025 (13)	−0.0056 (13)	0.0189 (15)
C32	0.0386 (17)	0.0442 (18)	0.0546 (19)	−0.0034 (13)	−0.0052 (14)	0.0128 (15)
C33	0.0424 (18)	0.0469 (19)	0.058 (2)	0.0129 (15)	0.0113 (15)	−0.0011 (15)
C34	0.0399 (16)	0.0527 (19)	0.0448 (16)	0.0029 (14)	0.0145 (13)	0.0049 (14)
C35	0.0438 (16)	0.0326 (14)	0.0489 (17)	0.0072 (12)	0.0091 (13)	−0.0060 (13)
C36	0.0355 (15)	0.0325 (15)	0.0509 (18)	0.0096 (12)	0.0148 (13)	−0.0055 (13)
C37	0.0339 (15)	0.0481 (18)	0.0450 (17)	0.0086 (13)	0.0091 (12)	−0.0081 (14)
C38	0.0371 (15)	0.0505 (18)	0.0397 (16)	0.0101 (13)	0.0089 (12)	−0.0016 (14)
C39	0.0318 (14)	0.0410 (15)	0.0368 (14)	0.0037 (11)	0.0032 (11)	−0.0065 (12)
C40	0.0330 (14)	0.0396 (16)	0.0428 (15)	−0.0076 (12)	0.0087 (12)	0.0002 (12)
C41	0.0309 (14)	0.0403 (15)	0.0461 (16)	−0.0013 (12)	0.0067 (12)	0.0028 (13)
C42	0.0381 (16)	0.0544 (19)	0.0459 (17)	0.0134 (14)	0.0035 (13)	0.0014 (15)
C43	0.0256 (13)	0.0537 (19)	0.0503 (17)	0.0067 (13)	−0.0089 (12)	0.0118 (15)
C44	0.0343 (16)	0.0488 (18)	0.0574 (19)	−0.0001 (13)	0.0177 (14)	0.0167 (15)
C45	0.0374 (16)	0.0502 (18)	0.0485 (17)	−0.0050 (14)	0.0057 (13)	0.0018 (15)
C46	0.0474 (17)	0.0502 (19)	0.0372 (16)	0.0021 (14)	0.0128 (13)	−0.0030 (14)
C47	0.054 (2)	0.0486 (19)	0.0398 (16)	0.0064 (15)	0.0113 (14)	−0.0024 (14)
C48	0.071 (2)	0.064 (2)	0.0406 (17)	0.0236 (19)	0.0238 (16)	0.0010 (16)
C49	0.0485 (18)	0.0510 (19)	0.0516 (18)	−0.0031 (15)	0.0174 (15)	0.0040 (15)
C50	0.0500 (19)	0.051 (2)	0.066 (2)	0.0079 (16)	0.0214 (16)	0.0120 (17)
C51	0.053 (2)	0.0357 (17)	0.0490 (17)	−0.0016 (14)	0.0170 (14)	−0.0021 (13)
C52	0.057 (2)	0.0485 (19)	0.0454 (18)	−0.0119 (15)	0.0151 (15)	−0.0010 (15)
Cl1	0.0543 (8)	0.0529 (8)	0.0400 (6)	0.0026 (6)	0.0133 (5)	−0.0035 (5)
Cl2	0.0559 (8)	0.0544 (8)	0.0461 (7)	0.0087 (6)	0.0155 (6)	−0.0008 (6)
Cl1'	0.0564 (13)	0.0522 (12)	0.0563 (12)	−0.0047 (9)	0.0214 (9)	0.0021 (10)
Cl2'	0.0534 (11)	0.0402 (10)	0.0516 (11)	0.0032 (8)	0.0135 (9)	−0.0007 (9)
Co1	0.03243 (18)	0.03119 (17)	0.04075 (19)	−0.00312 (17)	0.00836 (13)	0.00386 (18)
N1	0.0307 (13)	0.0365 (13)	0.0492 (16)	−0.0026 (10)	0.0118 (11)	0.0048 (12)
N2	0.0317 (12)	0.0365 (13)	0.0499 (14)	−0.0017 (10)	0.0074 (10)	−0.0021 (11)
N3	0.0432 (14)	0.0421 (14)	0.0432 (14)	0.0090 (11)	0.0033 (11)	0.0115 (11)
N4	0.061 (2)	0.0309 (14)	0.0445 (16)	−0.0045 (12)	0.0073 (14)	0.0030 (12)
N5	0.0311 (12)	0.0323 (12)	0.0497 (14)	−0.0009 (9)	0.0105 (10)	0.0081 (11)
N6	0.0590 (16)	0.0298 (12)	0.0426 (14)	−0.0034 (11)	0.0147 (12)	−0.0024 (11)
N7	0.0517 (17)	0.0637 (19)	0.0507 (16)	0.0008 (14)	0.0114 (13)	0.0039 (15)
N8	0.071 (2)	0.061 (2)	0.0559 (18)	0.0210 (17)	0.0214 (16)	−0.0034 (15)

### Geometric parameters (Å, °)

**Table:**

C1—N5	1.362 (4)	C32—C33	1.283 (5)
C1—C2	1.363 (5)	C32—H32A	0.9300
C1—H1A	0.9300	C33—C34	1.407 (5)
C2—C3	1.398 (5)	C33—H33A	0.9300
C2—H2A	0.9300	C34—C35	1.385 (4)
C3—C4	1.435 (5)	C34—H34A	0.9300
C3—H3A	0.9300	C35—N3	1.338 (4)
C4—C5	1.393 (5)	C35—C36	1.514 (4)
C4—H4A	0.9300	C36—N1	1.367 (4)
C5—N5	1.376 (4)	C36—C37	1.390 (5)
C5—C6	1.480 (4)	C37—C38	1.388 (4)
C6—N4	1.340 (4)	C37—H37A	0.9300
C6—C7	1.404 (5)	C38—C39	1.416 (4)
C7—C8	1.409 (5)	C38—C46	1.496 (5)
C7—H7A	0.9300	C39—C40	1.384 (4)
C8—C9	1.406 (5)	C39—H39A	0.9300
C8—C16	1.482 (5)	C40—N1	1.322 (4)
C9—C10	1.432 (5)	C40—C41	1.506 (4)
C9—H9A	0.9300	C41—N2	1.385 (4)
C10—N4	1.385 (4)	C41—C42	1.393 (4)
C10—C11	1.401 (5)	C42—C43	1.389 (5)
C11—C12	1.336 (4)	C42—H42A	0.9300
C11—N6	1.445 (4)	C43—C44	1.327 (5)
C12—C13	1.459 (5)	C43—H43A	0.9300
C12—H12A	0.9300	C44—C45	1.435 (5)
C13—C14	1.334 (5)	C44—H44A	0.9300
C13—H13A	0.9300	C45—N2	1.331 (4)
C14—C15	1.370 (5)	C45—H45A	0.9300
C14—H14A	0.9300	C46—C47	1.389 (5)
C15—N6	1.371 (4)	C46—C51	1.400 (5)
C15—H15A	0.9300	C47—C48	1.383 (5)
C16—C17	1.392 (5)	C47—H47A	0.9300
C16—C21	1.404 (5)	C48—C49	1.439 (5)
C17—C18	1.395 (5)	C48—H48A	0.9300
C17—H17A	0.9300	C49—C50	1.355 (5)
C18—C19	1.396 (6)	C49—C52	1.412 (5)
C18—H18A	0.9300	C50—C51	1.413 (5)
C19—C20	1.387 (6)	C50—H50A	0.9300
C19—C22	1.414 (5)	C51—H51A	0.9300
C20—C21	1.400 (5)	C52—N7	1.190 (4)
C20—H20A	0.9300	Co1—N4	1.980 (3)
C21—H21A	0.9300	Co1—N1	2.000 (3)
C22—N8	1.213 (5)	Co1—N3	2.089 (3)
C31—N3	1.332 (4)	Co1—N6	2.093 (3)
C31—C32	1.363 (5)	Co1—N2	2.105 (3)
C31—H31A	0.9300	Co1—N5	2.119 (3)
			
N5—C1—C2	123.5 (3)	C37—C36—C35	126.5 (3)
N5—C1—H1A	118.3	C38—C37—C36	120.8 (3)
C2—C1—H1A	118.3	C38—C37—H37A	119.6
C1—C2—C3	118.8 (3)	C36—C37—H37A	119.6
C1—C2—H2A	120.6	C37—C38—C39	117.8 (3)
C3—C2—H2A	120.6	C37—C38—C46	120.4 (3)
C2—C3—C4	120.3 (3)	C39—C38—C46	121.5 (3)
C2—C3—H3A	119.8	C40—C39—C38	118.8 (3)
C4—C3—H3A	119.8	C40—C39—H39A	120.6
C5—C4—C3	116.0 (3)	C38—C39—H39A	120.6
C5—C4—H4A	122.0	N1—C40—C39	122.3 (3)
C3—C4—H4A	122.0	N1—C40—C41	113.2 (3)
N5—C5—C4	123.8 (3)	C39—C40—C41	124.5 (3)
N5—C5—C6	111.7 (3)	N2—C41—C42	123.6 (3)
C4—C5—C6	124.4 (3)	N2—C41—C40	113.0 (3)
N4—C6—C7	120.8 (3)	C42—C41—C40	123.4 (3)
N4—C6—C5	115.4 (3)	C43—C42—C41	115.7 (3)
C7—C6—C5	123.7 (3)	C43—C42—H42A	122.1
C6—C7—C8	120.3 (3)	C41—C42—H42A	122.1
C6—C7—H7A	119.9	C44—C43—C42	123.1 (3)
C8—C7—H7A	119.9	C44—C43—H43A	118.5
C9—C8—C7	117.5 (3)	C42—C43—H43A	118.5
C9—C8—C16	121.5 (3)	C43—C44—C45	118.5 (3)
C7—C8—C16	121.0 (3)	C43—C44—H44A	120.8
C8—C9—C10	121.3 (3)	C45—C44—H44A	120.8
C8—C9—H9A	119.4	N2—C45—C44	121.6 (3)
C10—C9—H9A	119.4	N2—C45—H45A	119.2
N4—C10—C11	118.0 (3)	C44—C45—H45A	119.2
N4—C10—C9	117.5 (3)	C47—C46—C51	118.9 (3)
C11—C10—C9	124.4 (3)	C47—C46—C38	122.1 (3)
C12—C11—C10	128.4 (3)	C51—C46—C38	119.0 (3)
C12—C11—N6	121.3 (3)	C48—C47—C46	122.0 (3)
C10—C11—N6	110.4 (3)	C48—C47—H47A	119.0
C11—C12—C13	119.9 (3)	C46—C47—H47A	119.0
C11—C12—H12A	120.0	C47—C48—C49	118.0 (3)
C13—C12—H12A	120.0	C47—C48—H48A	121.0
C14—C13—C12	119.1 (3)	C49—C48—H48A	121.0
C14—C13—H13A	120.5	C50—C49—C52	122.8 (4)
C12—C13—H13A	120.5	C50—C49—C48	120.7 (3)
C13—C14—C15	119.6 (3)	C52—C49—C48	116.5 (3)
C13—C14—H14A	120.2	C49—C50—C51	120.1 (3)
C15—C14—H14A	120.2	C49—C50—H50A	120.0
C14—C15—N6	124.8 (4)	C51—C50—H50A	120.0
C14—C15—H15A	117.6	C46—C51—C50	120.2 (3)
N6—C15—H15A	117.6	C46—C51—H51A	119.9
C17—C16—C21	119.6 (3)	C50—C51—H51A	119.9
C17—C16—C8	119.3 (3)	N7—C52—C49	175.9 (4)
C21—C16—C8	120.9 (3)	N4—Co1—N1	170.50 (11)
C16—C17—C18	120.3 (4)	N4—Co1—N3	108.59 (12)
C16—C17—H17A	119.8	N1—Co1—N3	78.69 (11)
C18—C17—H17A	119.8	N4—Co1—N6	77.85 (11)
C17—C18—C19	119.8 (4)	N1—Co1—N6	108.82 (11)
C17—C18—H18A	120.1	N3—Co1—N6	89.60 (11)
C19—C18—H18A	120.1	N4—Co1—N2	95.44 (12)
C20—C19—C18	120.5 (4)	N1—Co1—N2	77.59 (11)
C20—C19—C22	119.5 (4)	N3—Co1—N2	155.92 (10)
C18—C19—C22	120.0 (4)	N6—Co1—N2	94.15 (11)
C19—C20—C21	119.7 (4)	N4—Co1—N5	77.46 (11)
C19—C20—H20A	120.1	N1—Co1—N5	96.34 (10)
C21—C20—H20A	120.1	N3—Co1—N5	93.23 (11)
C20—C21—C16	120.1 (4)	N6—Co1—N5	154.74 (10)
C20—C21—H21A	120.0	N2—Co1—N5	93.40 (10)
C16—C21—H21A	120.0	C40—N1—C36	120.8 (3)
N8—C22—C19	179.2 (4)	C40—N1—Co1	120.4 (2)
N3—C31—C32	125.0 (3)	C36—N1—Co1	117.9 (2)
N3—C31—H31A	117.5	C45—N2—C41	117.5 (3)
C32—C31—H31A	117.5	C45—N2—Co1	127.7 (2)
C33—C32—C31	118.5 (3)	C41—N2—Co1	114.74 (19)
C33—C32—H32A	120.8	C31—N3—C35	114.9 (3)
C31—C32—H32A	120.8	C31—N3—Co1	129.0 (2)
C32—C33—C34	122.1 (3)	C35—N3—Co1	116.1 (2)
C32—C33—H33A	118.9	C6—N4—C10	122.4 (3)
C34—C33—H33A	118.9	C6—N4—Co1	119.3 (2)
C35—C34—C33	115.0 (3)	C10—N4—Co1	117.6 (2)
C35—C34—H34A	122.5	C1—N5—C5	117.5 (3)
C33—C34—H34A	122.5	C1—N5—Co1	127.1 (2)
N3—C35—C34	124.3 (3)	C5—N5—Co1	115.35 (19)
N3—C35—C36	113.6 (3)	C15—N6—C11	115.1 (3)
C34—C35—C36	122.1 (3)	C15—N6—Co1	129.2 (2)
N1—C36—C37	119.5 (3)	C11—N6—Co1	115.61 (19)
N1—C36—C35	113.4 (3)		
			
N5—C1—C2—C3	−3.2 (5)	N3—Co1—N1—C40	174.6 (3)
C1—C2—C3—C4	1.8 (5)	N6—Co1—N1—C40	−99.8 (2)
C2—C3—C4—C5	−1.5 (5)	N2—Co1—N1—C40	−9.6 (2)
C3—C4—C5—N5	2.6 (5)	N5—Co1—N1—C40	82.5 (2)
C3—C4—C5—C6	179.5 (3)	N3—Co1—N1—C36	5.4 (2)
N5—C5—C6—N4	−4.4 (4)	N6—Co1—N1—C36	91.1 (2)
C4—C5—C6—N4	178.3 (3)	N2—Co1—N1—C36	−178.7 (2)
N5—C5—C6—C7	172.0 (3)	N5—Co1—N1—C36	−86.6 (2)
C4—C5—C6—C7	−5.3 (5)	C44—C45—N2—C41	0.7 (5)
N4—C6—C7—C8	0.1 (5)	C44—C45—N2—Co1	179.2 (2)
C5—C6—C7—C8	−176.1 (3)	C42—C41—N2—C45	0.4 (5)
C6—C7—C8—C9	−3.8 (5)	C40—C41—N2—C45	178.5 (3)
C6—C7—C8—C16	176.1 (3)	C42—C41—N2—Co1	−178.3 (3)
C7—C8—C9—C10	4.1 (5)	C40—C41—N2—Co1	−0.2 (3)
C16—C8—C9—C10	−175.7 (3)	N4—Co1—N2—C45	12.7 (3)
C8—C9—C10—N4	−0.9 (5)	N1—Co1—N2—C45	−173.9 (3)
C8—C9—C10—C11	175.5 (3)	N3—Co1—N2—C45	−163.8 (3)
N4—C10—C11—C12	−173.4 (3)	N6—Co1—N2—C45	−65.5 (3)
C9—C10—C11—C12	10.2 (5)	N5—Co1—N2—C45	90.4 (3)
N4—C10—C11—N6	5.8 (4)	N4—Co1—N2—C41	−168.8 (2)
C9—C10—C11—N6	−170.5 (3)	N1—Co1—N2—C41	4.7 (2)
C10—C11—C12—C13	176.4 (3)	N3—Co1—N2—C41	14.7 (4)
N6—C11—C12—C13	−2.7 (5)	N6—Co1—N2—C41	113.1 (2)
C11—C12—C13—C14	1.0 (6)	N5—Co1—N2—C41	−91.1 (2)
C12—C13—C14—C15	2.6 (6)	C32—C31—N3—C35	3.7 (5)
C13—C14—C15—N6	−4.8 (6)	C32—C31—N3—Co1	−177.9 (3)
C9—C8—C16—C17	30.1 (5)	C34—C35—N3—C31	−2.5 (5)
C7—C8—C16—C17	−149.8 (3)	C36—C35—N3—C31	178.9 (3)
C9—C8—C16—C21	−155.2 (3)	C34—C35—N3—Co1	178.9 (3)
C7—C8—C16—C21	24.9 (5)	C36—C35—N3—Co1	0.3 (3)
C21—C16—C17—C18	−1.2 (5)	N4—Co1—N3—C31	−7.7 (3)
C8—C16—C17—C18	173.6 (3)	N1—Co1—N3—C31	178.7 (3)
C16—C17—C18—C19	1.2 (6)	N6—Co1—N3—C31	69.4 (3)
C17—C18—C19—C20	−0.6 (6)	N2—Co1—N3—C31	168.7 (3)
C17—C18—C19—C22	−177.3 (4)	N5—Co1—N3—C31	−85.5 (3)
C18—C19—C20—C21	0.1 (6)	N4—Co1—N3—C35	170.7 (2)
C22—C19—C20—C21	176.8 (4)	N1—Co1—N3—C35	−3.0 (2)
C19—C20—C21—C16	−0.1 (6)	N6—Co1—N3—C35	−112.2 (2)
C17—C16—C21—C20	0.7 (5)	N2—Co1—N3—C35	−12.9 (4)
C8—C16—C21—C20	−174.0 (3)	N5—Co1—N3—C35	92.9 (2)
N3—C31—C32—C33	−4.0 (6)	C7—C6—N4—C10	3.4 (5)
C31—C32—C33—C34	2.9 (6)	C5—C6—N4—C10	179.9 (3)
C32—C33—C34—C35	−1.8 (5)	C7—C6—N4—Co1	−167.0 (3)
C33—C34—C35—N3	1.7 (5)	C5—C6—N4—Co1	9.5 (4)
C33—C34—C35—C36	−179.9 (3)	C11—C10—N4—C6	−179.6 (3)
N3—C35—C36—N1	4.0 (4)	C9—C10—N4—C6	−3.0 (5)
C34—C35—C36—N1	−174.6 (3)	C11—C10—N4—Co1	−9.1 (4)
N3—C35—C36—C37	−166.5 (3)	C9—C10—N4—Co1	167.5 (2)
C34—C35—C36—C37	14.9 (5)	N3—Co1—N4—C6	−97.3 (3)
N1—C36—C37—C38	3.2 (5)	N6—Co1—N4—C6	177.3 (3)
C35—C36—C37—C38	173.3 (3)	N2—Co1—N4—C6	84.2 (3)
C36—C37—C38—C39	−0.6 (5)	N5—Co1—N4—C6	−8.1 (3)
C36—C37—C38—C46	−175.7 (3)	N3—Co1—N4—C10	91.9 (3)
C37—C38—C39—C40	−0.6 (5)	N6—Co1—N4—C10	6.4 (2)
C46—C38—C39—C40	174.4 (3)	N2—Co1—N4—C10	−86.6 (3)
C38—C39—C40—N1	−0.7 (5)	N5—Co1—N4—C10	−178.9 (3)
C38—C39—C40—C41	−178.0 (3)	C2—C1—N5—C5	4.1 (5)
N1—C40—C41—N2	−7.2 (4)	C2—C1—N5—Co1	−174.9 (3)
C39—C40—C41—N2	170.3 (3)	C4—C5—N5—C1	−3.8 (4)
N1—C40—C41—C42	170.9 (3)	C6—C5—N5—C1	178.9 (3)
C39—C40—C41—C42	−11.6 (5)	C4—C5—N5—Co1	175.3 (2)
N2—C41—C42—C43	0.4 (5)	C6—C5—N5—Co1	−2.0 (3)
C40—C41—C42—C43	−177.5 (3)	N4—Co1—N5—C1	−175.7 (3)
C41—C42—C43—C44	−2.5 (5)	N1—Co1—N5—C1	11.6 (3)
C42—C43—C44—C45	3.6 (5)	N3—Co1—N5—C1	−67.4 (3)
C43—C44—C45—N2	−2.7 (5)	N6—Co1—N5—C1	−163.4 (3)
C37—C38—C46—C47	−28.6 (5)	N2—Co1—N5—C1	89.4 (3)
C39—C38—C46—C47	156.4 (3)	N4—Co1—N5—C5	5.2 (2)
C37—C38—C46—C51	148.7 (3)	N1—Co1—N5—C5	−167.5 (2)
C39—C38—C46—C51	−26.3 (5)	N3—Co1—N5—C5	113.6 (2)
C51—C46—C47—C48	−0.5 (5)	N6—Co1—N5—C5	17.6 (4)
C38—C46—C47—C48	176.8 (4)	N2—Co1—N5—C5	−89.6 (2)
C46—C47—C48—C49	−1.8 (6)	C14—C15—N6—C11	3.0 (5)
C47—C48—C49—C50	2.9 (6)	C14—C15—N6—Co1	−174.7 (3)
C47—C48—C49—C52	−177.9 (4)	C12—C11—N6—C15	0.8 (4)
C52—C49—C50—C51	179.1 (3)	C10—C11—N6—C15	−178.4 (3)
C48—C49—C50—C51	−1.7 (6)	C12—C11—N6—Co1	178.9 (3)
C47—C46—C51—C50	1.8 (5)	C10—C11—N6—Co1	−0.4 (3)
C38—C46—C51—C50	−175.6 (3)	N4—Co1—N6—C15	174.5 (3)
C49—C50—C51—C46	−0.7 (6)	N1—Co1—N6—C15	−12.5 (3)
C39—C40—N1—C36	3.4 (5)	N3—Co1—N6—C15	65.4 (3)
C41—C40—N1—C36	−179.1 (3)	N2—Co1—N6—C15	−90.8 (3)
C39—C40—N1—Co1	−165.5 (2)	N5—Co1—N6—C15	162.1 (3)
C41—C40—N1—Co1	12.1 (4)	N4—Co1—N6—C11	−3.2 (2)
C37—C36—N1—C40	−4.6 (5)	N1—Co1—N6—C11	169.8 (2)
C35—C36—N1—C40	−175.9 (3)	N3—Co1—N6—C11	−112.3 (2)
C37—C36—N1—Co1	164.5 (2)	N2—Co1—N6—C11	91.5 (2)
C35—C36—N1—Co1	−6.8 (3)	N5—Co1—N6—C11	−15.6 (4)
